# Colon cancer with extensive invasion of the abdominal wall treated with neoadjuvant chemotherapy and a free anterolateral thigh flap

**DOI:** 10.1186/s40792-022-01515-5

**Published:** 2022-08-19

**Authors:** Tsukasa Aritake, Akira Ouchi, Koji Komori, Takashi Kinoshita, Yusuke Sato, Ryota Nakamura, Keisuke Takanari, Hiroya Taniguchi, Kei Muro, Seiichi Kato, Tetsuya Abe, Seiji Ito, Yasuhiro Shimizu

**Affiliations:** 1 Department of Gastroenterological Surgery, Aichi Cancer Center Hospital, 1-1 Kanokoden, Chikusa, Nagoya, Aichi 464-8681 Japan; 2 Department of Plastic and Reconstructive Surgery, Aichi Cancer Center Hospital, 1-1 Kanokoden, Chikusa, Nagoya, Aichi 464-8681 Japan; 3 Department of Clinical Oncology, Aichi Cancer Center Hospital, 1-1 Kanokoden, Chikusa, Nagoya, Aichi 464-8681 Japan; 4 Department of Pathology and Molecular Diagnostics, Aichi Cancer Center Hospital, 1-1 Kanokoden, Chikusa, Nagoya, Aichi 464-8681 Japan

**Keywords:** Locally advanced colon cancer, Anterolateral thigh flap, Neoadjuvant chemotherapy

## Abstract

**Background:**

The treatment of locally advanced colon cancer is challenging, particularly when there is invasion of the abdominal wall. In such cases, balancing the securing of margins and sufficiently repairing abdominal wall defects is important, but difficult when the extent of invasion is large.

**Case presentation:**

A 34-year-old male was referred to our hospital with abdominal pain and diagnosed with obstructive transverse colon cancer. He had undergone ileo-sigmoid colostomy at his previous hospital. The tumor was massive and invaded the abdominal wall (maximum diameter: approximately 12 cm), and was accompanied by regional lymph node swelling. No distant metastasis was detected. We diagnosed the tumor as cT4bN2bM0 Stage IIIC locally advanced transverse colon cancer and planned neoadjuvant chemotherapy. After two courses of FOLFOXIRI (5-fluorouracil, leucovorin, oxaliplatin, and irinotecan), he developed an entero-cutaneous fistula due to tumor penetration and required emergency diverting ileostomy construction. After the procedure, contrast-enhanced computed tomography showed good tumor shrinkage. As a result, the planned chemotherapy was canceled and he underwent radical resection of the tumor. En bloc extended right hemicolectomy was performed with excision of the fistula, ensuring a sufficient margin. The post-excision defect at the anterior abdominal wall involved 11 × 16 cm of fascia and 6 × 9 cm of skin located in the middle of the abdomen. A free anterolateral thigh flap was harvested from the right thigh and vascular pedicle was anastomosed to the right gastroepiploic artery and vein. The fascia lata, which was included in the anterolateral thigh flap, was sutured onto the abdominal wall fascia as inlay fashion to reconstruct the abdominal wall defect. Histopathology revealed moderately differentiated adenocarcinoma of the colon with no tumor cells in the abdominal wall tissue [post-chemotherapeutic state, therapy effect: Grade 1b; Stage IIA (ypT3N0M0)]. All resected margins of the specimen were free from adenocarcinoma. He was discharged on postoperative day 16.

**Conclusion:**

We report a case of colon cancer extensively invading the abdominal wall, which was completely resected. The abdominal wall defect was reconstructed with a free anterolateral thigh flap after tumor shrinkage with neoadjuvant chemotherapy. We present an efficient strategy for managing locally advanced colon cancer with extensive abdominal wall invasion.

## Background

The treatment of locally advanced colon cancer is challenging, particularly for cases involving invasion of the abdominal wall. Such cases require carefully balancing the securing of margins and sufficiently repairing abdominal wall defects, which can be difficult when the extent of invasion is large. Primary closure, component separation, prosthetic mesh, and loco-regional, pedicled or free flaps can be used for reconstruction surgery [1]. However, when the defect is extensive, options are limited to fascia lata grafts and fasciocutaneous flaps.

Neoadjuvant chemotherapy (NAC) may be suitable for treating locally advanced colon cancer with extensive abdominal wall invasion, given problems with sizable abdominal wall defects. Significant improvements in downstaging and reducing the incomplete resection rate are reported benefits of neoadjuvant systemic therapy [2]. This suggests the possibility of applying NAC to locally advanced colon cancer with extensive invasion of the abdominal wall.

Here, we report a case of colon cancer extensively invading the abdominal wall. The tumor was completely resected and the abdominal wall was reconstructed with a free anterolateral thigh (ALT) flap after tumor shrinkage with NAC. We propose an efficient strategy for managing locally advanced colon cancer with extensive abdominal wall invasion.

## Case presentation

**Fig. 1:**
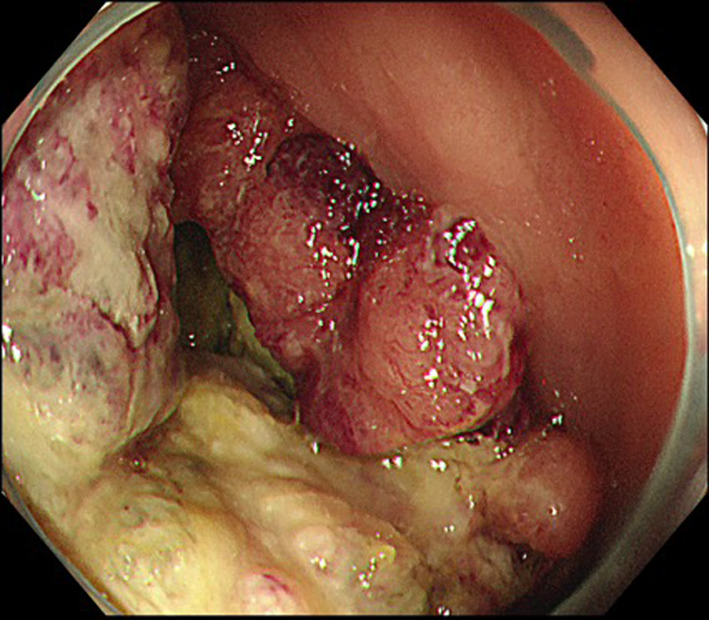
Colonoscopy findings at initial examination. Colonoscopy revealed a circumferential tumor in the transverse colon

**Fig. 2:**
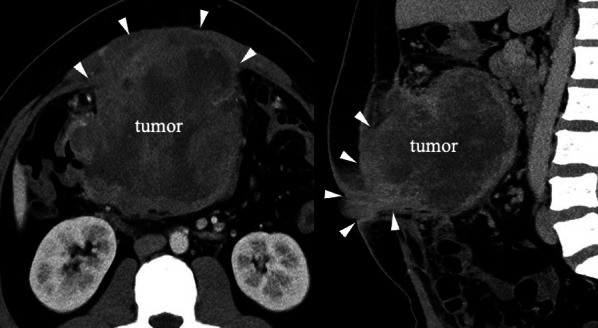
Contrast-enhanced CT scan findings at initial examination. Enhanced CT shows a large tumor with extensive direct invasion (approximately 12 cm, white arrowhead) of the anterior abdominal wall

A 34-year-old male was referred to our hospital with abdominal pain and diagnosed with obstructive transverse colon cancer. Colonoscopy revealed a circumferential tumor in the transverse colon, and histopathological examination revealed moderately differentiated adenocarcinoma (Fig. 1). He had undergone ileo-sigmoid colostomy at his previous hospital. The tumor markers such as CEA and CA19-9 were not elevated. Contrast-enhanced computed tomography (CT) showed a rapidly growing 12 cm tumor with extensive invasion of the anterior abdominal wall and regional lymph node swelling (Fig. 2). There was no evidence of distant metastasis. RAS/BRAF and MSI testing could not be performed due to insufficient tumor volume in biopsy tissue samples.

**Fig. 3:**
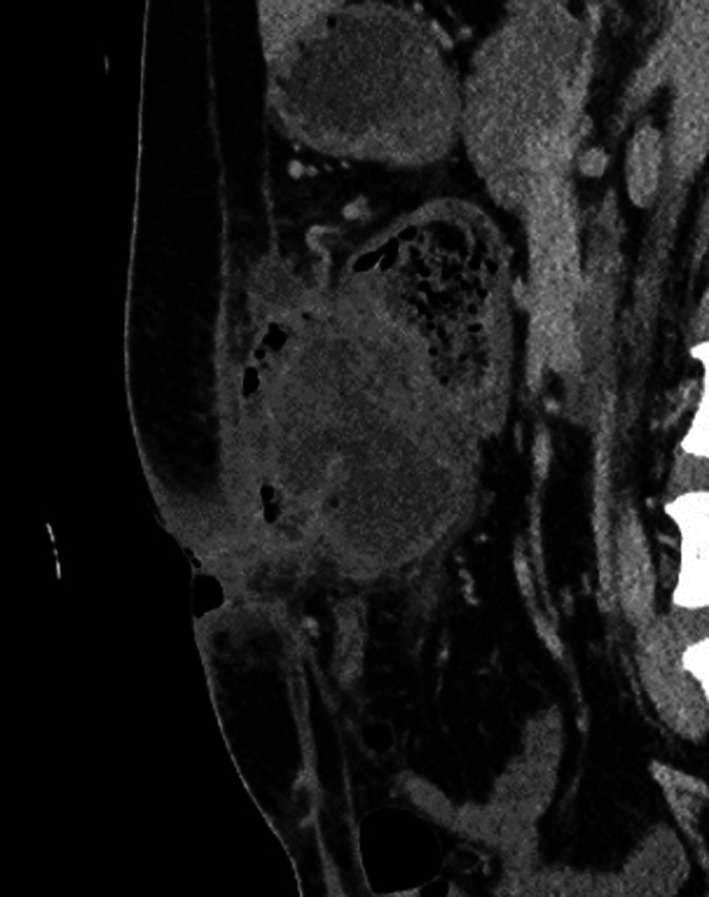
Contrast-enhanced CT scan findings after two courses of FOLFOXIRI. Enhanced CT shows an entero-cutaneous fistula due to tumor penetration of the abdominal wall

**Fig. 4:**
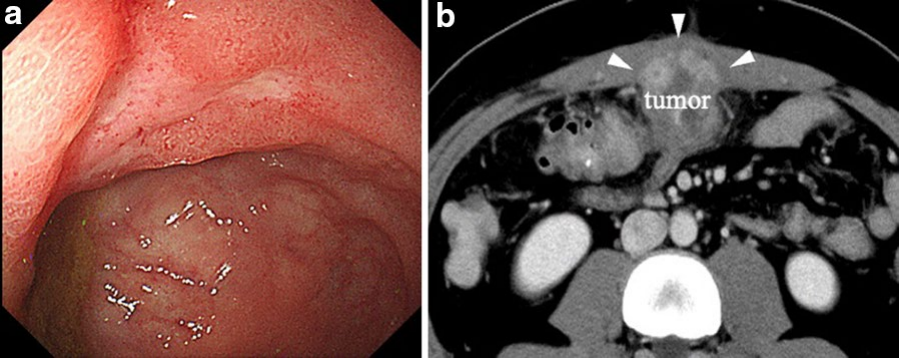
Colonoscopy and contrast-enhanced CT scan findings after emergent surgery. Colonoscopy reveals shrinkage of the tumor (**a**) and enhanced CT shows reduction of tumor invasion of the abdominal wall to about 5 cm (white arrowhead) (**b**)

We diagnosed the tumor as cT4bN2bM0 Stage IIIC locally advanced transverse colon cancer invading the abdominal wall according to the 8th Union for International Cancer Control (UICC) classification and initiated preoperative chemotherapy to reduce the extent of resection and reconstruction of the abdominal wall. Although four courses of the 5-fluorouracil, leucovorin, oxaliplatin, and irinotecan (FOLFOXIRI) regimen were initially planned, he developed an entero-cutaneous fistula due to tumor penetration of the abdominal wall (Common Terminology Criteria for Adverse Events, CTCAE-Grade 4) after two courses of FOLFOXIRI and required emergent construction of a diverting ileostomy (Fig. 3). Colonoscopy and contrast-enhanced CT after emergent surgery showed good tumor shrinkage (Fig. 4). According to Response Evaluation Criteria in Solid Tumors criteria (RECIST), the patient showed a partial response (PR) after preoperative chemotherapy (the reduction rate was about 60%), so the planned chemotherapy was canceled and he underwent radical resection of the tumor.

**Fig. 5:**
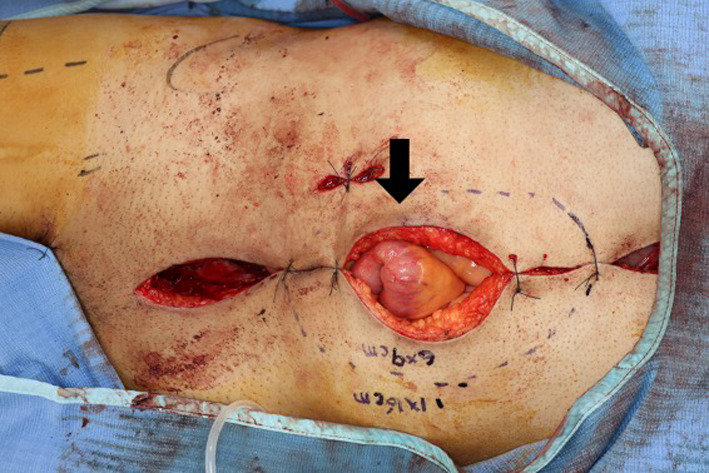
Intraoperative photograph. The post-excision defect at the anterior abdominal wall involved 11 × 16 cm of fascia and 6 × 9 cm of skin (black arrow)

**Fig. 6:**
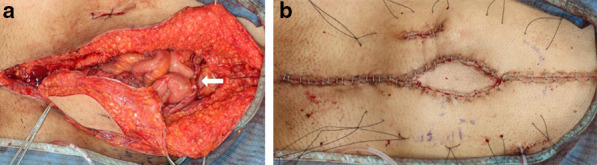
Intraoperative photograph. Intraoperative image showing that the ALT flap was supercharged using the right gastroepiploic artery and vein (white arrow) (**a**). Postoperative picture showing the flap sutured to the defect with a healthy color (**b**)

An upper midline incision was made with a boat-shaped abdominal wall resection. En bloc extended right hemicolectomy was performed with excision of the fistula, ensuring a sufficient margin. The ileostomy and ileo-sigmoid colostomy were also resected, and an ileo-ileostomy, ileo-transverse colostomy, and sigmoid-colocolostomy were performed with functional end-to-end anastomosis. The post-excision defect at the anterior abdominal wall involved 11 × 16 cm of fascia and 6 × 9 cm of skin (Fig. 5). A free ALT perforator flap, measuring 12 × 17 cm of fascia and 5 × 8 cm of overlying skin, was harvested from the right thigh. The descending branch of lateral circumflex femoral artery and vein were anastomosed to the right gastroepiploic artery and vein, and the fascia lata, which was included in the ALT flap, was sutured onto the abdominal wall fascia as inlay fashion to reconstruct the abdominal wall defect (Fig. 6a). The skin was closed, allowing for a complete tensionless defect cover (Fig. 6b).

Histopathology of the resected specimen revealed moderately differentiated adenocarcinoma of the colon with no tumor cells in the abdominal wall tissue but granulation and inflammatory cells, possibly due to the impact of neoadjuvant chemotherapy (post-chemotherapeutic state, therapy effect: Grade 1b). Lymphovascular invasion was observed, but no lymph node metastasis was found (out of 47 dissected lymph nodes). The tumor was diagnosed as Type 3, size 30 × 25 mm, Stage IIA (ypT3N0M0) according to the 8th UICC classification. All resected margins of the specimen were free from adenocarcinoma.

The patient developed intestinal paralysis, but underwent conservative treatment and was discharged on postoperative day 16. He had received the 5-fluorouracil, leucovorin, and oxaliplatin (FOLFOX) regimen as adjuvant postoperative chemotherapy, but developed severe mental retardation and dropped out of chemotherapy after the second course, and that is why genetic analysis using the excised specimens is still not available. There has been no evidence of disease 6 months postoperatively.

## Discussion

There are two points of clinical significance with the present case. First, an ALT flap was suitable for repairing the extensive abdominal wall defect after radical resection of locally invasive colon cancer. Second, NAC contributed to achieving radical resection of the massive tumor and reducing the extent of abdominal wall repair required.

Synthetic mesh, often selected for abdominal wall defects for which primary closure is difficult to perform, is unsuitable for clean-contaminated surgeries. In such cases, wide-coverage fasciocutaneous flaps are an option. The ALT flap, based on the lateral circumflex femoral system, was first described by Song et al. in 1984 as a free flap for late head and neck burn contractures [3]. In the present case, resection of the entero-cutaneous fistula with a sufficient margin resulted in an extensive full-thickness abdominal wall defect. The ALT flap was helpful for securing a sizeable cutaneous paddle and fascia lata with a long vascular pedicle, while ensuring minimal donor site morbidity [4]. While the pedicled ALT flap can cover lower abdominal defect, free ALT flap can cover any level of the abdominal defect. In the current case, we chose to use ALT as free flap because the defect located in the middle of the abdomen, which was not reachable with pedicled ALT flap. In abdominal reconstruction using a free flap, both extraperitoneal and intraperitoneal vessels can be utilized as recipients [5, 6]. In our case, right gastroepiploic vessels were used as recipients because intra-abdominal vessels were readily available due to open surgery. Moreover, utilizing intra-abdominal vessels have additional advantage that fascial fixation is more reliable by suturing the entire circumference of the flap, which is not achievable with pedicled ALT.

There are no specific guidelines for managing locally advanced colon cancer [7]. However, NAC may be a promising option given its potential to promote tumor regression. While NAC is not the standard treatment for colon cancer and only a few perioperative and preoperative chemotherapy trials have been conducted [2], a recent FOXTROT trial reported a higher complete resection rate with sufficient margins using NAC compared to adjuvant chemotherapy (96% vs. 80%) without an increase in postoperative complications [8]. In the trial, NAC also resulted in pathological downstaging [8]. In the present case, NAC reduced the extent of abdominal wall resection, which led to a reliable radical resection and safe reconstruction with the ALT flap. In addition, there is also currently no consensus on the optimal neoadjuvant regimen for the treatment of locally advanced colon cancer. However, FOLFOXIRI was expected to have a higher response rate than doublet regimens even in tumors with unknown genomic status as in this case [9].

Disadvantages of NAC have also been reported. For instance, Wadhwani et al. presented a similar case of locally advanced colon cancer in which postchemotherapy necrosis resulted in tissue fragility and hemorrhage in the tumor that required surgery as an oncological emergency [7]. The present case also required urgent ileostomy due to tumor penetration, suggesting the same risk of perforation or bleeding. On the other hand, F. Ali et al. reported a case of locally advanced sigmoid colon cancer with abdominal wall invasion in which a full-thickness abdominal wall defect measuring 11 × 13 cm after radical resection was successfully repaired with pedicled ALT flap, without performing NAC [1]. While NAC for locally advanced colon cancer has several advantages, the indication should be carefully considered for each case by examining the patient’s general condition and disease progression.

## Conclusion

This case report presents an efficient strategy for managing locally advanced colon cancer with extensive abdominal wall invasion. Multidisciplinary therapy and collaboration were successful in treating far advanced colon cancer.

## Data Availability

Data sharing is not applicable since no datasets were generated or analyzed during the present study.

## Abbreviations

NAC: Neoadjuvant chemotherapy
ALT: Anterolateral thigh
CT: Computed tomography
UICC: Union for International Cancer Control
FOLFOXIRI: 5-Fluorouracil, leucovorin, oxaliplatin, and irinotecan
